# A 4-year outbreak of MRSA ST72-MRSA-IV *spa* type t1597 in a surgical high dependency unit in Ireland linked to repeated healthcare worker recolonisation

**DOI:** 10.1016/j.infpip.2024.100421

**Published:** 2024-11-15

**Authors:** Deirdre Brady, Grainne Brennan, Brian O'Connell, Ruth Buckley, Marie Brennan, Maria Lenehan, Jincy Jerry, Lars Nolke, Seyed Hossein Javadpour, Margaret M. Hannan, Breda Lynch, Maureen Lynch

**Affiliations:** a Department of Clinical Microbiology, Mater Misericordiae University Hospital, Dublin, Ireland; b UCD School of Medicine, UCD Health Sciences Centre, University College Dublin, Dublin, Ireland; c National MRSA Reference Laboratory, St. James' Hospital, Dublin, Ireland; d Department of Clinical Microbiology, School of Medicine, University of Dublin, Trinity College, St. James's Hospital, Dublin, Ireland; e Department of Clinical Microbiology, St. James' Hospital, Dublin, Ireland; f Department of Quality, Safety and Risk, Mater Misericordiae University Hospital, Dublin, Ireland; g Department of Nursing Quality, Mater Misericordiae University Hospital, Dublin, Ireland; h Department of Occupational Medicine, Mater Misericordiae University Hospital, Dublin, Ireland; i Department of Infection Prevention and Control, Mater Misericordiae University Hospital, Dublin, Ireland; j Department of Cardiothoracic Surgery, Mater Misericordiae University Hospital, Dublin, Ireland

**Keywords:** MRSA, Outbreak, Healthcare worker, Colonisation, Screening, Cardiac surgery

## Abstract

**Background:**

Patients undergoing cardiac surgery are identified as high risk for *Staphylococcus aureus* infection, including MRSA. An outbreak of MRSA was identified when two patients experienced MRSA infection concurrently in a cardiothoracic high dependency unit with uncommon detection of MRSA previously and an established screening programme.

**Methods:**

An outbreak control team was convened and interventions applied including refresher training in hand and environmental hygiene, review of practice with regard to aseptic access of medical devices and consideration of antibiotic use in the unit. MRSA isolates were referred to the Irish National MRSA Reference Laboratory where *spa* typing assigned all isolates to t1597 and whole genome sequencing assigned them to multilocus sequence type ST72-MRSA-IV. Recovery of this strain from only this unit in Ireland and infrequent reporting in Europe prompted staff MRSA screening with two staff members found to harbour the outbreak strain. Despite successful decolonisation, recolonisation and further transmission to patients occurred.

**Conclusions:**

In the clinical unit in which this outbreak occurred, the usual control measures to prevent spread of MRSA were in place. Recent Joint Healthcare Infection Society and Infection Prevention Society Guidance does not recommend routine staff screening for MRSA but does support its consideration in an outbreak of an unusual strain. In total, 9 patients and 2 staff were affected by this outbreak. There were 4 infections and 3 deaths. Sustained outbreak closure was necessary to protect certain national clinical programmes and was achievable only when colonised staff were no longer working in the unit.

## Background

Methicillin-resistant *Staphylococcus aureus* (MRSA) is a well-recognised pathogen in healthcare associated infection (HAI). Multiple interventions are used in hospitals to prevent spread of the organism and individual patient infection, including but not limited to hand hygiene practice, transmission-based precautions, screening of groups of patients deemed to be at higher risk of MRSA infection, decolonisation of colonised patients, and care with insertion and maintenance of indwelling devices [1,2]. Patients undergoing cardiac surgery are identified as high risk for *Staphylococcus aureus* infection [2], including methicillin-sensitive *S. aureus* (MSSA) or MRSA.

## Setting

The cardiothoracic high dependency unit (CTHDU) was a 10 bed unit located in a large tertiary academic teaching hospital. Three of the beds were ensuite positive pressure ventilated lobby (PPVL) rooms. The remaining 7 beds were arranged as bays in a single row which in 2019 were divided only by walls between the bays. Subsequently had front walls retrofitted for additional protection at the onset of the COVID-19 pandemic. A pre-operative screening program for *S. aureus* and MRSA, on admission and weekly thereafter was well-established. Prior to the identification of this outbreak, patient acquisition of MRSA within the unit had been sporadic since its opening in 2013**.**

## Outbreak investigation

**Figure 1:**
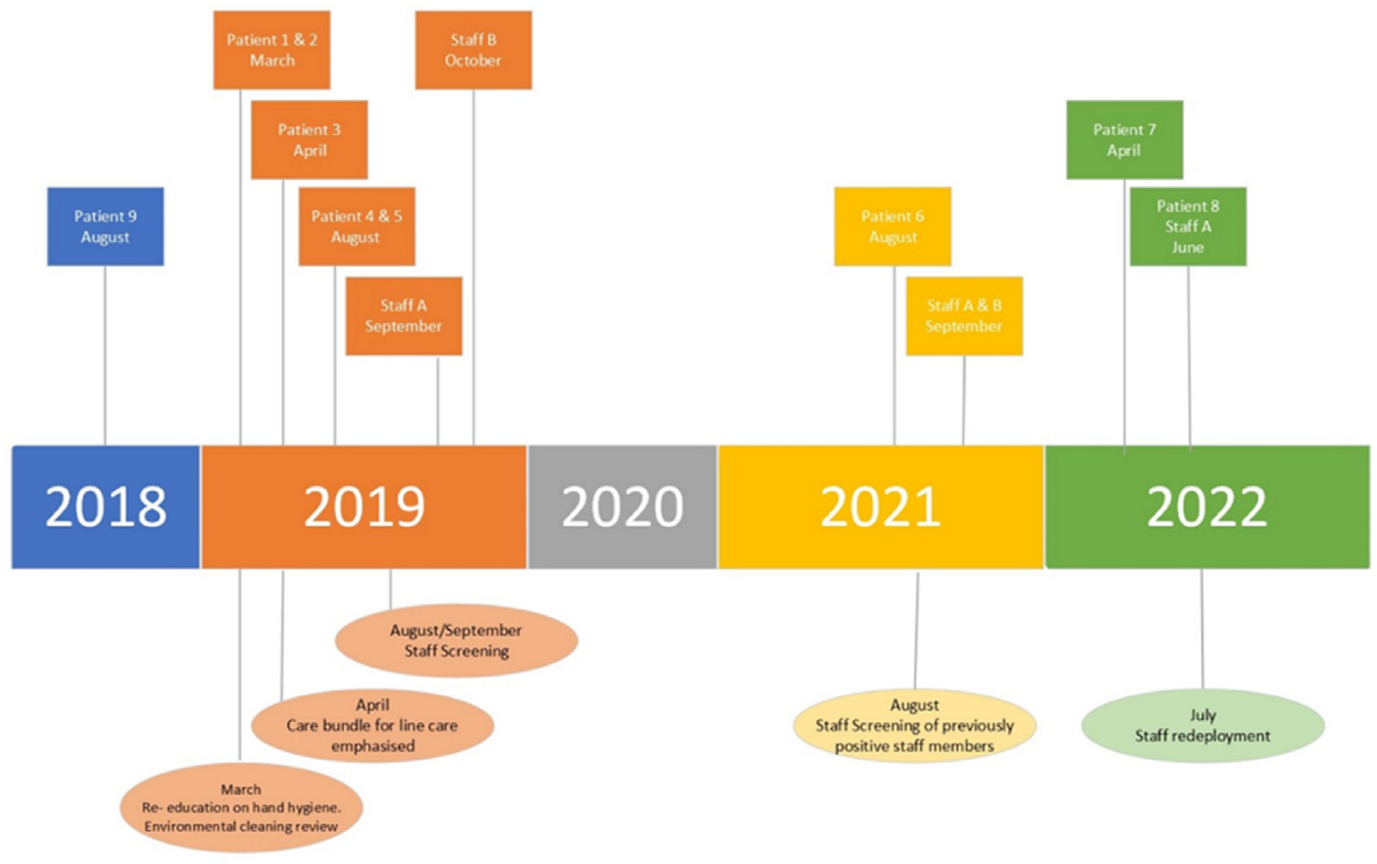
Timeline of outbreak and implementation of interventions applied.

In March 2019, MRSA was recovered from two patients accommodated in CTHDU within two days of each other (Figure 1). Both patients had undergone screening as part of pre-operative assessment and post-operatively during admission with negative results but subsequently MRSA was recovered from blood (Patient 1) and sputum (both patients). Both patients died from their infection. As the patient reservoirs were no longer in the unit and regular screening was in place for patients, a full outbreak control team was not convened immediately but the ward was informed of the cases and educated about hand and environmental hygiene.

Approximately one week after the identification of the initial cases, MRSA was recovered from a third patient during routine MRSA screening. At this time an Outbreak Control Team was convened to include nursing and medical staff from the affected area, members of the Infection Prevention and Control Team led by the Consultant Microbiologist, representatives from Quality and Patient Safety, General Services (Hygiene), Occupational Medicine, Estates and Facilities and the hospital executive management team. Concurrently, a Serious Incident Review process was commenced because two patients had died.

In August 2019, two further patients were identified as being newly colonised with MRSA (4^th^ and 5^th^cases) and so it was agreed to undertake MRSA screening of staff working within the unit. This was carried out confidentially by the Department of Occupational Medicine with individual staff members' concerns addressed. Not all relevant staff members were reached for screening. For those staff who consented, nasal screens were cultured on selective MRSA agar with sensitivities performed on positive samples including mupirocin. Two staff members (Staff members A and B) were found to be colonised with the outbreak strain and underwent successful decolonisation with negative results on repeat screening.

In August 2021, two years after the initial outbreak was first identified, one patient was found to be colonised with the outbreak MRSA strain during routine weekly screening. At this time staff members A and B who were previously colonised with the outbreak strain underwent re-screening and were both found to have become recolonised. Decolonisation was again offered and was successful.

Approximately eight months later in April 2022, a patient who had been accommodated in CTHDU 5 days earlier was admitted to ICU for management of severe healthcare associated pneumonia (HAP). MRSA was recovered from a sputum sample. This patient died with pneumonia. MRSA *spa* typing revealed this isolate to be the outbreak strain suggesting that it was likely a staff member was shedding MRSA.

In June 2022 a further patient was colonised with MRSA on weekly screening, without signs or symptoms of infection (8^th^ patient). Screening of staff was implemented and there was good compliance. Staff members with at least a weekly commitment to the unit were invited for screening. In total 50 staff members were screened and included nursing (21/50), doctors (17/50) and health and social care professionals (9/50). At this time, one staff member (Staff member A) was identified as colonised with the outbreak strain. Staff member B was no longer working in the unit at this time.

Following a retrospective review of all isolates recovered in the previous 10-year period, a 9th patient was identified as having had MRSA recovered from sputum in 2018. This patient had been in the CTHDU six-months before the outbreak was identified. The MRSA had not been identified as CTHDU-acquired and was not associated with any contemporaneous cases to suggest an outbreak. This patient may in fact have been the source case of this outbreak or simply the first patient affected by a staff member shedding. A review of the clinical notes identified that patient as having HAP but responding to appropriate broad spectrum antibiotics, thus reducing the mortality rate to 75% patients with clinical infection.

## Patient characteristics – the first three patients at the time of outbreak identification

**The index case-Patient 1** was a 65-year old female who had originally presented with acute mitral valve papillary muscle rupture and underwent mechanical mitral valve replacement approximately eight weeks prior to MRSA infection. The patient had a prolonged and complicated postoperative critical care requirement, initially in the intensive care unit (ICU), moving to the high dependency unit (HDU) and the cardiothoracic surgical high dependency unit (CTHDU) on days 26 and 32 post-op, respectively. The patient developed a gastrointestinal bleed and sepsis in CTHDU 53 days postoperative and required transfer back to the ICU for management of shock due primarily to sepsis. MRSA was recovered from various sites including blood cultures, sputum, central vascular catheter tip and skin swabs taken at the time of clinical deterioration. The patient was treated broadly for healthcare associated infection including vancomycin and clindamycin to treat MRSA and underwent removal of potentially infected devices such as peripherally inserted central catheter (PICC) and central vascular catheter (CVC) but remained bacteraemic over four days. Although her bacteraemia cleared by day five, the impact of the infection was too great and the patient died 65 days post-surgery.

**Patient 2** was a 64-year old female with a background of hypertension, type 2 diabetes mellitus, ischaemic heart disease with previous myocardial infarction and peripheral vascular disease. The patient was transferred to the hospital for consideration for coronary artery bypass grafting and aortic valve replacement which she underwent approximately 10 weeks prior to developing an MRSA infection. A postoperative ICU stay was complicated by a re-sternotomy on day 11 for delayed cardiac tamponade, as well as a healthcare associated pneumonia (HAP) and acute kidney injury requiring haemodialysis. She was transferred to CTHDU on day 17 post-op and her stay was complicated by *Serratia marcescens* bacteraemia, likely PICC-related and candidaemia, likely CVC-related. On the 69^th^ post-operative day the patient developed new symptoms and signs consistent with HAP. Microbiological examination of sputum culture identified *S. aureus* and treatment including vancomycin to treat potential MRSA infection was started empirically. The patient continued to deteriorate but had an agreed ceiling of care to the level of CTHDU and she died on day 70 post surgery. MRSA was confirmed on sputum culture post-mortem.

**Patient 3** was a 55-year old male with end-stage heart failure, awaiting heart transplant, due to non-ischaemic dilated cardiomyopathy who underwent placement of left and right ventricular assist devices (LVAD, RVAD), as a bridge to transplant, approximately ten weeks prior to MRSA being recovered in the groin on routine screening. This 10-week interval included 25 days in ICU postoperatively and a return to theatre for the removal of the RVAD. Prior to the recovery of MRSA, other post-op complications included HAP and *Enterococcus faecalis* bacteraemia which was presumed PICC-related. This patient did not develop MRSA infection, was decolonised and subsequently had a successful cardiac transplant and was discharged home.

A brief summary of all nine patients affected during the whole outbreak is provided in the supplementary material.

## Laboratory Investigations

**Antimicrobial susceptibility testing** on *S. aureus* isolates recovered from screening and clinical samples was carried out on the Vitek®2 system (bioMérieux, Marcy l'Étoile, France) with isolates susceptible to ciprofloxacin and exhibiting resistance to penicillin, flucloxacillin, erythromycin and tetracycline only. Isolates were referred to the National MRSA Reference Laboratory (NMRSARL) for further epidemiological typing where they underwent *spa* typing and whole genome sequencing (WGS). *spa* typing assigned all isolates to t1597 while WGS assigned the isolates to multilocus sequence type ST72-MRSA-IV. All isolates were assigned to clonal complex (CC) 8. Virulence genes identified included the Enterotoxin gene cluster comprised of *seg, sei, sem, sen, seo* and *seu* along with Enterotoxin C. All isolates were negative for PVL, exfoliative toxins and toxic shock toxin *tsst*.

**Figure 2:**
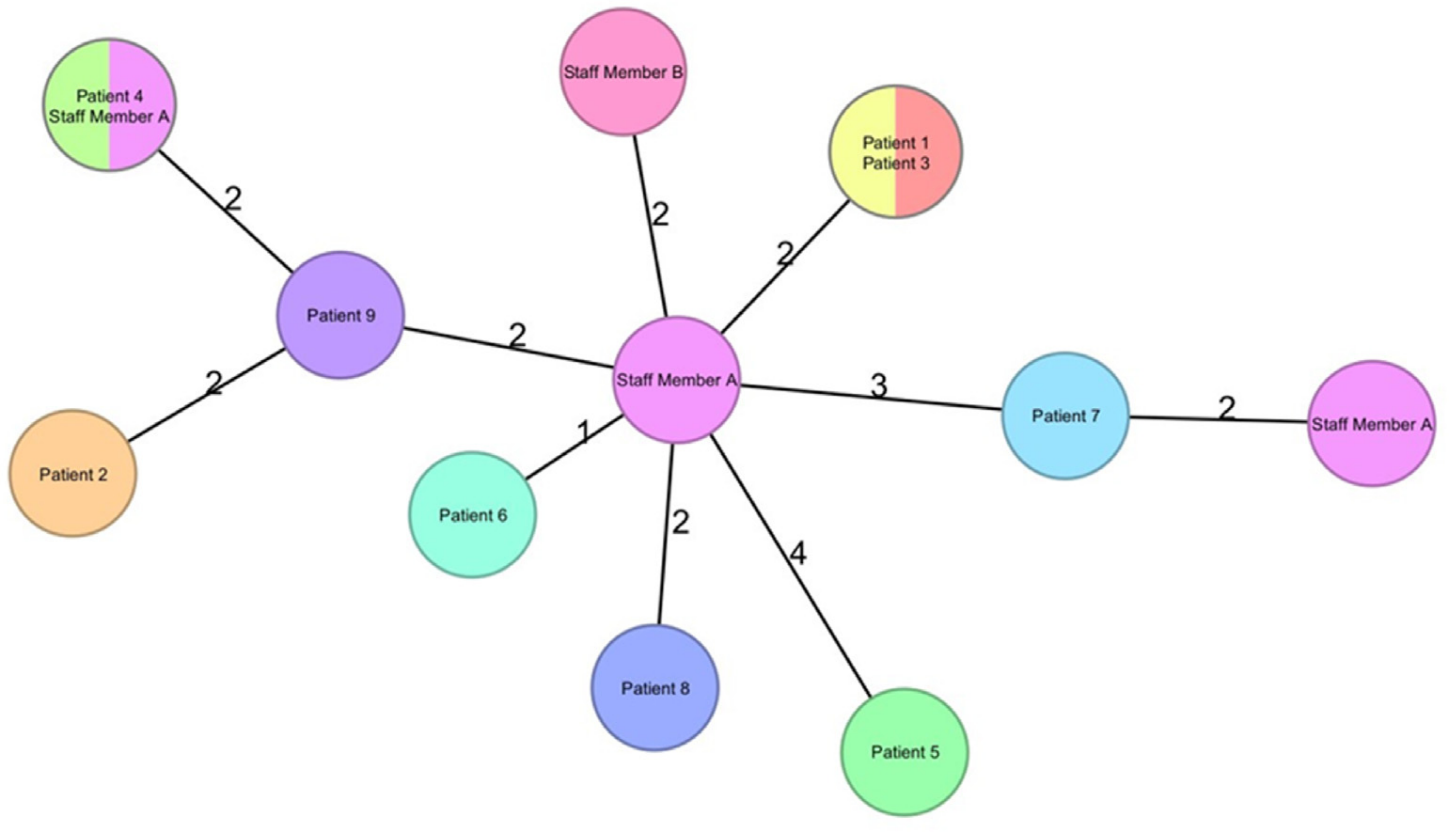
Distance based minimum spanning tree for 13 isolates based on core genome MLST of 1,861 genes, pairwise ignoring missing values generated by SepSphere ™ (Jünemann *et al.*, 2013). Nodes are coloured by patient/staff member.

A minimum spanning tree was constructed based on the core genome MLST (1861 genes) where the cluster distance threshold of 24 was applied [3]. All isolates clustered together in a single cluster differing from each other by ≤4 allelic differences (Figure 2).

## Interventions

Within the unit, hand hygiene practice and environmental hygiene practices were reviewed and refresher education delivered to all staff. In addition, practice with regard to aseptic access of intravenous lines and devices was reviewed. A care bundle for central vascular access devices had been recently introduced in the hospital and implementation was ongoing at the time of this outbreak however its use in the unit was not fully established (See supplementary material for care bundle details). Antimicrobial usage within the unit was considered. Antimicrobial usage was guided by a weekly Multidisciplinary Team Meeting (MDT) with a clinical microbiologist, antimicrobial pharmacist and a member of the cardiothoracic team.

Two staff members were found to harbour the outbreak strain and underwent successful decolonisation with negative results on repeat screening. Following recolonisation, both were once again successfully decolonised. Since the second episode of recolonization (3^rd^ total episode with MRSA detected) the remaining colonised staff member was no longer working in cardiothoracic care.

At the time of writing, no MRSA outbreak cases have been identified in the unit or the hospital since June 2022 and so the outbreak is considered closed.

## Discussion

We describe a prolonged outbreak caused by ST72-MRSA-IV in a CTHDU where nine patients and two staff members were either colonised or infected with the strain. Three patients died due to MRSA infection while staff members became recolonised on at least two occasions.

MRSA is a common healthcare-associated pathogen. In Ireland the rate of healthcare associated MRSA staphylococcal infections has been declining from 38% in 2000 to 10.4% in 2022. Since the early 2000s, ST22-MRSA-IV has been associated with approximately 80% of MRSA blood stream infections (BSI) however more recently it has been reported that the epidemiology of MRSA in Ireland is changing. ST22-MRSA-IV now accounts for only 45% of MRSA BSI with increased diversity seen throughout the remaining MRSA [[4], [5], [6], [7]] population. In addition, a number of community associated MRSA strains have been associated with outbreaks in Irish healthcare settings [3,8,9].

Whole genome sequencing assigned this strain to ST72-MRSA-IV and *spa* type t1597. Isolates assigned to this *spa* type have not been recovered elsewhere in Ireland (NMRSARL, unpublished data) and are also not frequently reported elsewhere in Europe. In contrast, this strain is a major CA-MRSA strain in Korea with increasing prevalence in the community and hospitals during the past 20 years [10]. Of note this strain is frequently associated with skin and soft tissue infections, HAP and community acquired necrotising pneumonia [[11], [12], [13]]. It is likely that the risk factors of patients in the cardiothoracic unit in terms of underlying cardiac and respiratory conditions, transcutaneous and respiratory devices and long recovery periods after major complex surgery contributed to the severity of infection and fatal outcome. Here, as this strain was associated with a disproportionate number of deaths (three deaths out of nine identified patients to date and 3/4 of those with clinical infection), sustained closure of the outbreak was necessary and was only achieved after both colonised staff members were no longer working in the unit. Contact tracing for a link to Korea was not completed as only one staff member was available by the time the isolate was identified as common in that country.

Whilst the adherence to good hand hygiene practices in reducing the spread of MRSA is widely accepted, the role of shedding by colonised HCW is not as clearly understood [14]. The low number of patients affected since the opening of the outbreak, along with favourable audit results suggested that hand hygiene within the unit was good. The Irish National MRSA Guideline does not recommend routine HCW screening for MRSA but does suggest it be considered during an outbreak of an unusual strain [2]. This is reiterated in the more recent Joint Healthcare Infection Society and Infection Prevention Society Guidance [1]. The exclusivity of this strain to one ward in one centre helped to inform the screening process, with recognition among staff that concerns about patient safety in the unit could lead to the temporary cessation of a number of national clinical programs until it was resolved. As the MRSA was an unusual strain type, the possibility that it was introduced to the unit by a visitor or member of staff was considered. Visiting was reviewed but deemed unlikely on the basis of the timing of the positive results. When the question of staff screening was initially raised it was acknowledged that approximately 1 in 170 healthy Irish adults was colonised with MRSA [15] and as such around 20 healthcare workers (HCW) at our centre could have been colonised at any given time and we do not screen HCWs in any department. It is expected that hand hygiene prevents spread to patients.

In the unit in which this outbreak occurred, the usual control measures to prevent spread of MRSA between patients or from staff to patient were in place but it must be acknowledged that there are instances when patients in this unit deteriorate quickly and an immediate emergency response from multiple staff is required. Thus there were opportunities for hygienic practice to be imperfect due to the need to rapidly preserve the life of the patient. Some patients required the assistance of multiple staff members simultaneously to move e.g. 6–7 members of staff may be required to move a patient who has a VAD. As such breaches in hand hygiene may have occurred. If one staff member is colonised with MRSA there is potential for spread during such a breach. In addition, many patients in the unit had indwelling devices such as CVC or PICC. At the time of this outbreak, hospital-wide implementation of a care bundle for central vascular access devices was in progress, and not fully established.

It was noted that no incidences of MRSA were detected during the period August 2019–June 2021. Ongoing review of hand hygiene practices within the unit showed good compliance but this may also be related to the COVID-19 pandemic. It has been reported elsewhere that enhanced IPC measures implemented in response to the COVID-19 pandemic reduced the occurrence of MRSA infections in non-COVID-19 patients [16,17]. Universal wearing of facemasks in delivering clinical care was introduced in Ireland under an instruction by the Chief Clinical Officer in early April 2020 and this was adopted in our hospital and continued until May 2023. There were no compliance data mask wearing and its impact on preventing MRSA transmission before June 2021 but it was noted anecdotally that compliance throughout the hospital was better prior to universal vaccination for SARS-CoV-2 and dropped as the numbers of severe COVID-19 infections and deaths also fell. Mask wearing has not been introduced as an additional measure within the care bundle for central vascular access devices though this may change if evidence in favour of this practice emerges following the pandemic.

This outbreak spanned several years with just nine patients affected. The evidence that transmission had been rare due to an overall high standard of practice in the unit was a welcome positive for unit staff. Management of the outbreak has been used as an educational tool to drive adherence to best practice in prevention of healthcare-associated infection.

## CRediT author statement

Deirdre Brady: Conceptualisation, Investigation, Resources, Writing – Original Draft, Writing - Review and Editing, Visualisation, Supervision, Project Administration.

Grainne Brennan: Validation, Formal Analysis, Investigation, Resources, Data Curation, Writing – Review and Editing, Visualisation.

Brian O'Connell: Validation, Formal Analysis, Investigation, Writing – Review and Editing, Visualisation.

Ruth Buckley: Investigation, Resources, Writing – review and Editing.

Marie Brennan: Investigation, Resources, Writing – review and Editing.

Maria Lenehan: Investigation, Resources, Writing – review and Editing.

Jincy Jerry: Investigation, Resources, Writing – Review and Editing.

Lars Nolke: Writing – Review and Editing.

Seyed Hossein Javadpour: Writing – Review and Editing.

Margaret M Hannan: Writing – Review and Editing.

Breda Lynch: Investigation, Writing- Review and Editing.

Maureen Lynch: Writing – Review and Editing, Supervision.

## Informed consent

Informed consent was not gained from patients involved in this outbreak. All patients were treated according to clinical judgement and infection control practices in order to treat them and control the outbreak according to local guidelines. Patients did not undergo randomisation or intervention for the purpose of this report. Data has been analysed and presented anonymously.

## Ethics

Ethical approval was not required.

## Funding

This research did not receive any specific grant from funding agencies in the public, commercial, or not-for-profit sectors.

## Conflict of interest

The authors have no conflict of interest to declare.
