# Supplementary material for: A 4-year outbreak of MRSA ST72-MRSA-IV spa type t1597 in a surgical high dependency unit in Ireland linked to repeated healthcare worker recolonisation
Source: Infect Prev Pract. 2024 Nov 15;7(1):100421. doi: 10.1016/j.infpip.2024.100421 (PMC11647124; doi:10.1016/j.infpip.2024.100421)
Supplement: Supplementary file 1 [file mmc1.pdf]

Patient Summary

	2018	2019/1	2019/2	2019/3	2019/4	2019/5	2021	2022/1	2022/2
Gender	Male	Female	Female	Male	Male	Male	Male	Male	Male
Underlying disease prognosis	Life limiting cardiac disease	Life limiting cardiac disease	Life limiting cardiac disease	Life limiting cardiac disease	Life limiting lung disease	Life limiting cardiac disease	Life limiting cardiac disease	Life limiting cardiac disease	Life limiting cardiac disease
Surgery since admission prior to MRSA detection	CABG	Mechanical MVR and re-sternotomy for clot removal	CABG and bioprosthetic AVR and re-sternotomy for tamponade	VAD placement and two further visits for adjustment / RVAD removal	Lung volume reduction surgery, VATS	Dental treatment, LVAD, RVAD insertion and removal	CABG and bioprosthetic AVR	Ileofemoral endarterectomy , CABG	Pericardectomy
Infected or colonised	**Infected**	**Infected**	**Infected**	Colonised	Colonised	Colonised	Colonised	**Infected**	Colonised
Site of MRSA	Sputum	Sputum, Sacrum, **Blood**, CVAD tip	Sputum	Groin	Nose	Nose, Throat	Grouped screen	Grouped screen, Sputum	Grouped screen
Outcome	Transferred back to referring hospital	Deceased	Deceased – MRSA diagnosed post-mortem	Received heart transplant; transfer back to referring hospital	Transferred back to referring hospital		Discharged to convalescence	Deceased	Discharged home

MRSA Decolonisation Regimen

Decolonisation Regimen

- Isolate patient in a single room with contact precautions
- Bactroban nasal ointment TDS X 5 days to both nostrils
- Chlorhexidine gargles or throat sprays BD X 5 days
- Chlorhexidine bath daily X 5 days

Day 1	Day 2	Day 3	Day 4	Day 5
Body	Body and Hair	Body	Body and Hair	Body

- Sputum/wound positive for MRSA – commence decolonisation as above and discuss with microbiology team
- 2 days rest
- Rescreen X 3 at 48 hour intervals
