# Supplementary material for: A 4-year outbreak of MRSA ST72-MRSA-IV spa type t1597 in a surgical high dependency unit in Ireland linked to repeated healthcare worker recolonisation
Source: Infect Prev Pract. 2024 Nov 15;7(1):100421. doi: 10.1016/j.infpip.2024.100421 (PMC11647124; doi:10.1016/j.infpip.2024.100421)
Supplement: Supplementary file 2 [file mmc2.pdf]

Attach Patient Addressograph

Patient Name: ____________________

MRN: ____________________

D.O.B: ____________________

Ward/Department: ____________________

Central Venous Access Device (CVAD)
Care Bundle Management Tool

Nursing Diagnosis: Risk for Surgical Site Infection
Goal: Prevention of catheter related blood stream infections (CRBSI)
Nursing Diagnosis: Risk for Vascular Trauma
Goal: Maintain patency of CVAD

CVAD INSERTION RECORD
Complete on insertion or on receipt of patient care.

Location of Insertion:
- Emergency Department: ☐
- Interventional Radiology: ☐
- Operating Theatre: ☐
- ICU: ☐
- Other: __________ *(Name location)*

Date of Insertion: __________

Type of CVAD *(Tick as appropriate)*
- Central venous catheter (CVC) ☐
- Permcath ☐
- PICC ☐
- Hickman ☐
- Port-a-cath ☐
- Other: __________ *(Insert name)*

CVAD Number of Lumens:
1 /2 /3 /4 /5 /6 /7 / Port *(Circle as appropriate)*

Site of Insertion *(Tick as appropriate)*
- Subclavian ☐
- Jugular ☐
- Femoral ☐
- Left side ☐
- Right side ☐
- Arm ☐
- Other: __________ *(Specify)*

Indication for use *(Tick as appropriate)*
- Intravenous therapy ☐
- Parenteral Nutrition ☐
- Vasopressor / inotrope therapy ☐
- Dialysis ☐
- Planned diagnostic procedure ☐
- Chemotherapy ☐
- Other: __________ *(Specify)*

Parenteral Nutrition
Designated lumen labelled Yes / No
__________ *(Specify lumen)*

CVAD patient education
Yes / No *(Circle as appropriate)* Date: __________

CVAD REMOVAL RECORD
Complete on removal of CVAD

Removal Date: __________

Reason for Removal *(Tick as appropriate)*
- Treatment completed ☐
- Infection suspected ☐
- Lumens blocked ☐
- Requires new CVAD ☐
- Patient RIP ☐
- Other: __________ *(Specify)*

Tip sent for C & S: Yes / No *(Circle as appropriate)*

NURSING INTERVENTIONS
Commence the care bundle for each device, on insertion or on receipt of patient care.
Complete the CVAD management care bundle at least 12 hourly.

Assess Indication for CVAD
- Verify the clinical indication and requirement for the CVAD are still valid through a daily review with the medical team (Critical Care RANP if CVC).

Hand Hygiene
- Perform Hand Hygiene before and after accessing or dressing a CVAD as per the 5 Moments for Hand Hygiene.

CVAD Insertion Site Assessment
- Inspect the CVAD insertion site for evidence of: redness, tenderness, pain, swelling, drainage, suture integrity, position.
- Ensure the CVAD insertion site is covered with an intact and transparent semipermeable dressing.
- Change the CVAD dressing every 7 days or earlier if the dressing becomes loose or dislodged or insertion site requires cleansing.

Assess Patient for Evidence of Infection
- Suspect infection if there are clinical signs of inflammation (redness, tenderness, pain, swelling, drainage) at the CVAD exit site.
- Suspect infection if there are clinical signs of sepsis (fever, tachypnoea, tachycardia, hypotension).
- If infection is suspected, inform the patient's medical team (Critical Care RANP if CVC) and discuss the need for removal of the CVAD.

Aseptic Non-Touch Technique (ANTT™)
- Where the CVAD access hub is a luer lock needleless connector and it is not disconnected from the CVAD apply clean gloves and utilise strict Aseptic Non-Touch Technique (ANTT™) when accessing a CVAD
- Where the CVAD access hub is a luer lock needleless connector and it is disconnected from the CVAD apply sterile gloves and utilise strict Aseptic Non-Touch Technique (ANTT™) when accessing a CVAD
- Utilise sterile equipment when accessing a CVAD.

Decontaminate CVAD Access Hubs
- Use a single-use application of 2% chlorhexidine gluconate in 70% isopropyl alcohol wipe (or 70% isopropyl alcohol wipe for patients with sensitivity to Chlorhexidine) to decontaminate each CVAD access hub and allow to air dry for 30 seconds prior to each CVAD hub access.

CVAD Lumen Patency
- Flush all CVAD lumens every 24 hours at a minimum if not in continuous use and check integrity of CVAD.
- When accessing a CVAD lumen for any purpose check for blood return to establish patency.
- Flush each lumen of the CVAD with a minimum of 10 mL of 0.9% NaCl using a push-pause method and end with positive pressure at the last 1 mL while clamping the lumen to maintain patency.
- CVAD device should flush easily and blood return should be free – flowing. If occlusion is suspected consult section 8.12 in policy for management.

P05040

Attach Patient Addressograph

Patient Name: ______________________________

MRN: ______________________________

D.O.B: ______________________________

Ward/Department: ______________________________

CVAD Management Care Bundle Tool

Complete every 12 hours at a minimum. Put a tick ✓ for a yes response, ✗ for a no response, ***N/A*** *for not applicable, in each box.*

Date	Time	Indication for CVAD still valid	Hand Hygiene performed before & after all CVAD access procedures	CVAD insertion site inspected and dressing intact.	Patient assessed for evidence of infection	Aseptic Non-Touch Technique (ANTT™) utilised during all access procedures	CVAD access hubs decont-aminated prior to all access procedures	CVAD lumens aspirated and flushed	Signature

Variance

Date	Time	**Variance –** ***record any variance from the bundle and nursing interventions i.e. change of CVAD dressing, change of CVAD access hub leur-lock needleless connector, evidence of site inflammation, removal of CVAD.***	Signature

Reference: MMUH (2018) Policy for the nursing management of Central Venous Access Devices (CVAD) including peripherally inserted central catheters (PICC), Hickman lines and Port-a-caths in the Mater Misericordiae University Hospital (MMUH). Available at: Q-Pulse PPC- NURS-147.
